# Validation of the Breastfeeding Experience Scale in a Sample of Iranian Mothers

**DOI:** 10.1155/2014/608657

**Published:** 2014-05-19

**Authors:** Forough Mortazavi, Seyed Abbas Mousavi, Reza Chaman, Ahmad Khosravi

**Affiliations:** ^1^Department of Midwifery, Faculty of Nursing and Midwifery, Sabzevar University of Medical Sciences, Sabzevar 9613873136, Iran; ^2^Research Center of Psychiatry, Golestan University of Medical Sciences, Golestan 4918936316, Iran; ^3^School of Medicine, Yasuj University of Medical Sciences, Yasuj 7591741418, Iran; ^4^Center for Health Related Social and Behavioral Sciences Research, Shahroud University of Medical Sciences, Shahroud 3614773955, Iran

## Abstract

*Objectives*. The aim of this study was to validate the breastfeeding experience scale (BES) in a sample of Iranian mothers. *Methods*. After translation and back translation of the BES, an expert panel evaluated the items by assessing the content validity ratio (CVR) and content validity index (CVI). 347 of mothers visiting health centers completed the Farsi version of the BES in the first month postpartum. Exploratory factor analysis (EFA) and confirmatory factor analysis (CFA) were performed to indicate the scale constructs. Reliability was assessed by Cronbach's alpha coefficient. *Results*. CVR and CVI scores for the BES were 0.96 and 0.87, respectively. Cronbach's alpha coefficient for the BES was 0.83. The results of the EFA revealed a new 5-factor model. The results of the CFA for the BES indicated a marginally acceptable fit for the proposed model and acceptable fit for the new model (RMSEA = 0.064, SRMR = 0.064, *χ* ^2^/df = 2.4, and CFI = 0.95). Mothers who were exclusively breastfeeding at the first month postpartum had less breastfeeding difficulties score (30.3 ± 7.6) than mothers who were on partial breastfeeding (36.7 ± 11.3) (*P* < 0.001). *Conclusions*. The Farsi version of the BES is a reliable and valid instrument to assess postpartum breastfeeding difficulties in Iranian mothers.

## 1. Introduction 

Breastfeeding brings benefits for both mother and baby [1]. The World Health Organization recommended exclusive breastfeeding (EBF) for all infants up to six months [2].

Iranian government has encouraged breastfeeding since the 1990s and significant success has been achieved, so that the rate of any breastfeeding at one year of age has reached 90% [3]. However, the rate of EBF is decreasing [4]. EBF rates at 4 and 6 months of age at national level averaged 56.8% and 27.7% [3]. Results of a study in Kerman, Iran, showed that partial breastfeeding rate at the end of the first month postpartum averaged 60% [5].

Breastfeeding difficulties are common. Previous studies in Iran, Sweden, and Canada revealed that 34%, 27%, and 87% of mothers in early postpartum period reported a breastfeeding difficulty, respectively [6, 7]. Sore nipple, engorgement, fatigue, feeling tired, difficult latching on, fussy baby, and insufficient supply of breast milk were the common breastfeeding problems [5].

Most breastfeeding difficulties are a relatively normal experience [8]; however, due to wide range of severity, they can be very stressful [9] and have been a risk factor for breastfeeding discontinuation in different studies [6, 10]. A study in the USA showed that mothers who had experienced breastfeeding difficulties in the first month postpartum had a higher risk for discontinuing full breastfeeding before 6 months and any breastfeeding before 12 months [11]. On the other hand, studies showed that support during early postpartum period was associated with increased EBF duration [12]. It is therefore necessary that breastfeeding difficulty be measured routinely during early postpartum period; however, due to the lack of a valid instrument for this purpose, for use in primary health care settings, most mothers with breastfeeding difficulty in the postpartum period remain undiagnosed. It is therefore important to validate an appropriate instrument for the task of measuring breastfeeding difficulty in the postpartum period. Since the breastfeeding experience includes multiple factors related to infant and mother, it is recommended to measure difficulties more multidimensionally and in the form of a continuous variable [9, 13, 14].

The instrument that was developed and validated to measure common breastfeeding difficulty in the form of a continuous variable in the postpartum period is the breastfeeding experience scale (BES) [15]. The first 18 items of the BES measure the severity of breastfeeding difficulties. The validity and reliability of this instrument have been examined and confirmed [13, 16, 17]. The aim of this study, therefore, was to translate and investigate the reliability and validity of the BES in a sample of Iranian mothers. To our best knowledge, no study has validated the BES in mothers in Iran.

## 2. Materials and Methods

This study was part of a larger study on the assessment of breastfeeding attrition prediction tools and was conducted on 358 pregnant women in late pregnancy of which 347 mothers visited 10 health clinics affiliated to Shahroud University of Medical Sciences in Shahroud, Iran, in 2011, for postpartum visit. The sampling method was convenient and the inclusion criteria were as follows: having a healthy baby and the ability to read and write. The subjects were informed that their participation was voluntary and all their information will be kept confidential. The Ethics Committee of the Shahroud University of Medical Sciences approved the study protocol (Approval no. 900.02). We obtained permission to use the BES from the author. The mothers completed the Farsi version of the BES and GHQ-28 at the end of the first and second month postpartum, respectively. Infant-feeding practice was evaluated at the end of the first month postpartum using the BES.

### 2.1. Instruments

Participants completed a questionnaire consisting of sociodemographic and obstetrical information (age, level of education, employment status, family income, parity, mode of delivery, and infant birth weight) at the 2-week postpartum visit. In addition, intention to breastfeed was assessed by a question using a 5-point numerical rating scale in late pregnancy (1: definitely breastfeed, 6: definitely not breastfeed).

#### 2.1.1. GHQ-28

The General Health Questionnaire (GHQ-28) is one of the screening tools used in epidemiological studies of psychiatric disorders [18]. It contains 28 questions in four subscales: somatic symptoms, anxiety and insomnia, social dysfunction, and severe depression. Each item is scored on a 4-point Likert scale ranging from zero to 3. The total score ranges from 0 to 84, where a higher score indicates lower psychological well-being. The validity of the Farsi version of the instrument has been supported in previous study [19]. The clinical cut-off point for screening general health in Iran has been estimated at 24, which represent probable psychological health problems requiring more evaluation.

#### 2.1.2. Breastfeeding Experience Scale (BES)

Breastfeeding experiencescale (BES) [17] is a questionnaire that consists of 30 items. The first 18 items measure presence or absence and severity of common breastfeeding difficulties in the early postpartum period. Scores range from “not at all" (1) to “unbearable" (5). The total score ranges from 18 to 90, with a higher score representing increased problem severity. The scale includes five subscales as follows: breast concerns (three items: sore nipples, cracked nipples, and breast infection), process concerns (five items: leaking breasts, baby reluctant to nurse due to sleepiness, breast engorgement, baby nursing too frequently, and feeling very tired), mechanic concerns (five items: baby having sucking difficulty, baby having difficulty in latching on, baby reluctant to nurse due to fussiness, feeling tense and overwhelmed, and difficulty in positioning baby), milk insufficiency concerns (three items: worry about not having enough milk, worry about baby's weight gain, and worry that baby was not getting enough milk), and social concerns (two items: feeling embarrassed when nursing and difficulty in combining work and breastfeeding). Content validity and internal consistency of this scale (alpha = 0.76) were demonstrated during early development of the BES [17]. In another study, the internal consistency of the questionnaire at 3 and 6 weeks postpartum was 0.79 and 0.72, respectively [20]. Also, in a study on 31 mothers with mastitis, the *α*-coefficient for the 18 items was 0.81 [16]. The last 12 items of the BES assess whether breastfeeding was continued, formula was added or substituted breast milk, how often formula was introduced, and what breastfeeding difficulties were related to mother's weaning decision in case of early weaning.

### 2.2. Statistical Analysis

Data analyses were conducted by SPSS version 18 (SPSS Inc., Chicago, IL, USA) and LISREL version 8.80 (Scientific Software International Inc., 2007). The reliability of the Farsi version of the BES was assessed by Cronbach's alpha coefficient, alpha if item deleted, interitem, and item-total correlation coefficients. Cronbach's alpha values >0.6, item-total correlation coefficients >0.20, and interitem correlations coefficients <0.80 and higher than zero were regarded as acceptable. Cronbach's alpha values <0.5 were regarded as unacceptable. An item was considered for removal if its item-total correlation coefficient was lower than 0.2, provided that its deletion led to an increase of more than 0.1 in Cronbach's alpha coefficient [21].

Exploratory factor analysis (EFA) was conducted utilizing principal component analysis with varimax rotation. Criteria for retaining factors and items were having eigenvalues >1 [22] and item loading ≥0.3 [23], respectively. Confirmatory factor analysis (CFA) was conducted by structural equation modeling. The method of estimation was weighted by the least squares. The asymptotic covariance matrix was considered as a weighted matrix. The input matrix was covariance matrix of data. Relative chi-squares <5.00, a CFI value >0.90, a RMSEA value of <0.08 [24], and a SRMR value of <0.08 [25] were considered as acceptable model fit. RMSEA and SRMR values greater than 0.10 justify rejecting the model [26].

Concurrent validity was examined by calculating Pearson's correlation coefficients between the BES and GHQ28. Correlation coefficients higher than 0.50 were considered indicative of good concurrent validity in similar instruments. For known group comparison, we compared the mean score of the BES in primiparous and multiparous mothers. For predictive validity, we compared the mean score of the BES in exclusive, predominant, and partial breastfeeding mothers using ANOVA test. Paired* t*-test was performed to compare the BES scores in primiparous and multiparous mothers.

### 2.3. Process of Translation and Cultural Adaptation

First, two specialists in English language translated the BES separately. Then, we discussed differences between the two translated versions and created the final version. Finally, a Ph.D. in English language who had not read the original version of the instrument translated the Farsi version into English. We compared the two English versions and found no discrepancy. Few minor revisions were done.

#### 2.3.1. Content Validity

Content validity was based on the judgment of experts that items and questions in an instrument were essential, relevant, and appropriate to the target culture. Therefore, the purpose of this step was to ensure that the Farsi version of the BES was clear and culturally relevant. Both qualitative and quantitative methods were applied [27]. In the qualitative phase, an expert panel consisted of 10 faculty members and specialists of reproductive health and pediatrics, gynecologists, nutritionists, epidemiologists, psychologists, and midwives who had paper in breastfeeding and evaluated grammar, wording, and scaling of the questionnaire. Four experts argued that the rate of introduction of water-based fluids was high in our population. Therefore, we added one question and changed two questions to cover the introduction of different water-based fluids. The Q22 “are you using any fluids (boiled water, sugar water, herbal teas) to feed your baby?" was added and Q23 was changed to assess how often they used fluids. In order to determine content validity ratio (CVR), we chose Lawshe approach [28]. Experts assessed essentiality of each item for the Iranian culture. They assessed the necessity of the items using a three-point rating scale: (a) not necessary, (b) useful, but not essential, and (c) essential. The CVR for every item was calculated using formula CVR = [*n* − (*N*/2)]÷(*N*/2) (*N *= the total number of experts and* n *= the number of experts who had chosen the (c) option for each particular item). We computed a CVR for the total scale. According to the Lawshe table, an acceptable CVR value for 10 experts is 0.62. No item had a CVR less than 0.62. The mean CVR for the total scale was 0.96, indicating a satisfactory content validity.

Then, the BES was given again to the experts to express their ideas about clarity, simplicity, and relevancy of each item in a 4-point Likert scale (from a: not relevant, not simple, and not clear to d: very relevant, very simple, and very clear). The content validity index for every item was calculated by dividing the total number of experts by the number of experts who had chosen the (c) or (d) option for each particular item (15). We calculated the CVI for relevancy, clarity, and simplicity of every item, according to the 10 experts' views for each item. Polit and Beck recommended 0.80 as the acceptable lower limit for the CVI value [29]. The mean CVI for the total scale was 0.87.

#### 2.3.2. Pilot Study

In the pilot study, we asked 20 low educated breastfeeding multiparas visiting two health centers to fill out the translated BES to assess how understandable are the items and questions and how long the BES takes to be completed. After the mothers individually completed the BES, we conducted face-to-face interviews to determine if they felt difficulty or ambiguity in responding to the items. Most mothers indicated that the questionnaire was easy to read and understand. However, some suggested changing item “difficulty in combining work and breastfeeding” to “difficulty in combining homemaking or work outside and breastfeeding” and suggested a better idiomatic equivalence for cracked nipple.

## 3. Results 

### 3.1. Subjects

The median age, educational level, and monthly family income of mothers were 26.1 years, 11 years, and 4 million RLS, respectively. Mode of delivery for 49% of mothers was vaginal. At the end of the first months postpartum the number of mothers who were on exclusive, predominant, and partial breastfeeding was 115 (33.1%), 202 (58.2%), and 30 (8.6%), respectively. Among mothers who were on partial breastfeeding, seven mothers started introducing formula within the first week postpartum and 20 mothers introduced formula every day. Among mothers who were on predominant breastfeeding, 153 mothers started introducing fluids within the first three days postpartum and 36 mothers introduced fluids every day. There was no early weaning at the end of the first month postpartum. All items of the scale have been answered.

### 3.2. Validity 

#### 3.2.1. Exploratory Factor Analysis

Exploratory factor analysis (EFA) was used to investigate factor structure of the BES within the sample. The Kaiser-Meyer-Olkin (KMO) measure of sampling adequacy was 0.817 and Bartlett's test of sphericity was significant (*χ* ^2^ = 1856, *P* < 0.001), indicating that the variables correlated with one another. Factor analysis yielded five factors ((1) mother concern, (2) insufficient milk concern, (3) baby concern, (4) breast concern, and (5) process concern) with eigenvalues ≥1, which explained 58.57% of total variance. Only “insufficient milk concern” factor was the same factor that the BES developer found. Factors 1 and 3 emerged. One item was added to the “breast concern” factor and 3 items were excluded from the “process concern” factor which Wambach found. The percentage variance and eigenvalues explained for rotated factors as well as the factor loading after rotation of each item are presented in Table 1. All items had factor loadings more than 0.396.

#### 3.2.2. Confirmatory Factor Analysis

**Figure 1:**
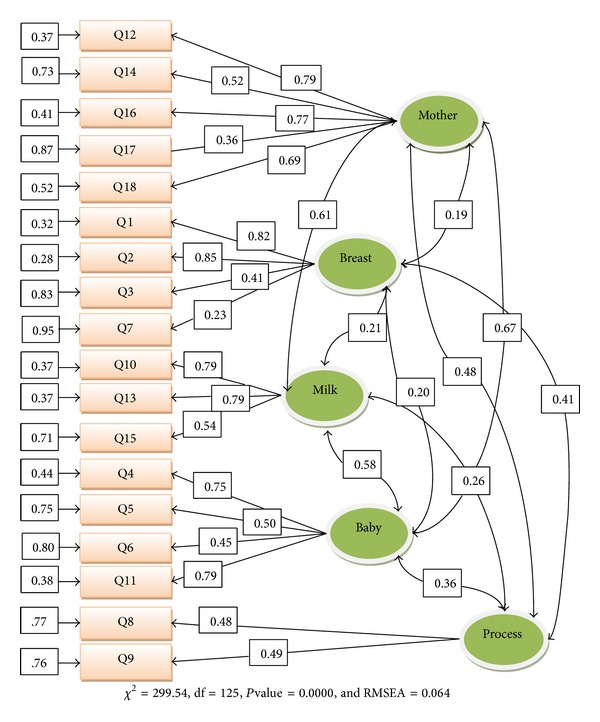
CFA of the new five-factor model of the BES (the item numbers refer to question numbers in the original questionnaire).

We used CFA to assess how well the model extracted by EFA and the factor structure suggested by previous study fitted the observed data. The results of the CFA for the two five-factor structures for the BES indicated a marginally acceptable fit for the proposed model and acceptable fit for the new model (RMSEA = 0.064, SRMR = 0.064, *χ* ^2^/df = 2.4, and CFI = 0.95). All parameters were significant (*T *value > 2). Results are shown in Table 2 and Figure 1. Factor load of items was 0.23 to 0.85.

#### 3.2.3. Concurrent Validity

We assumed that the mothers with breastfeeding difficulties would experience psychological problems. The correlation coefficients between the BES and GHQ-28 were 0.54, indicating moderate relationships (*P* < 0.001). In addition, we expected that maternal education and intention to breastfeed were negatively correlated with the BES scores. The results showed that mothers with higher education experienced higher breastfeeding difficulties (*R* = 0.26 and *P* = 0.037). As we had been expecting, mothers who were more determined to breastfeed in late pregnancy had lower breastfeeding difficulties (*R* = −0.146 and *P* = 0.006). However, both correlation coefficients were low.

#### 3.2.4. Known Group Comparison

In this study, we assumed that multiparous mothers had lower BES scores than primiparous mothers. There were 200 multiparas and 147 primiparas in our sample. Results showed that the mean BES score in multiparous mothers (30.1 ± 6.4) was lower than that of primiparous mothers (32.3 ± 9.7) (*t* = 2.53 and *P* = 0.012).

#### 3.2.5. Predictive Validity

We also evaluated the construct validity by determining the predictive validity of the instrument. We assumed that mothers with less breastfeeding difficulties would exclusively breastfeed their baby. Scores were compared by infant-feeding method at the first month postpartum. There were significant differences in breastfeeding difficulties score between mothers who were exclusively breastfeeding (30.3 ± 7.6), predominant breastfeeding (31.3 ± 8.3), and partial breastfeeding (36.7 ± 11.3) (*F* = 6.79, *P* < 0.001). The Scheffe testrevealed that mothers who were exclusively breastfeeding had less breastfeeding difficulties scores than mothers who were on partial breastfeeding.

### 3.3. Reliability

Table 3 shows Cronbach's alpha coefficients for the five subscales of the BES as originally assigned by Wambach [13].  Table 4 shows descriptive statistics and alpha Cronbach coefficients for the BES subscales as extracted by the EFA. The values of alpha Cronbach coefficients for subscales of the BES were higher for primiparas than for multiparas for both five-factor models. Both models had one factor, which did not meet the Cronbach's alpha criteria for reliability. Interitem correlation coefficients for each subscale as assigned by this study were 0.06 to 0.71. All corrected item-total correlation coefficients for each subscale were 0.22 to 0.63. Deleting each item only resulted in a slight reduction in Cronbach's alpha coefficient (0.01–0.05) except item 7 (breast infection) and item 15 (worry about baby's weight gain) that increased Cronbach's alpha coefficient (0.05 and 0.04, resp.).

## 4. Discussion and Conclusions 

This study was the first to describe the validity and reliability of this instrument in mothers in another language. The BES assesses the breastfeeding difficulties, practices, and outcomes. Both CVR and CVI were satisfactory, indicating that the content of the BES is congruent with the Iranian culture. All items have been answered. This demonstrates that the instrument was understandable to the mothers in this study. The results indicate that the Farsi version of the first 18 items of the BES is a reliable and valid instrument for measuring and quantifying breastfeeding difficulties in mothers.

The EFA extracted five factors, which jointly explained 58.57% of variances. These factors were not completely the same factors, which Wambach found [15]. Two new factors (mother concern and baby concern) emerged and the number of items of two factors (breast concern and process concern) changed. CFA marginally confirmed the five-factor structure of the BES proposed by Wambach in our population. Although the value of RMSEA was higher than 0.08, it was lower than 0.1 which did not justify rejecting the model proposed by Wambach [26]. The results of the CFA for the new five-factor structure of the BES were satisfactory, indicating a good fit to the data. The standardized loadings represent the correlation between each observed variable and the corresponding factors. There was only one item with factor load lower than 0.3. Assessment of parameters revealed that all of them are significant (*T *value > 2), indicating that each item is significantly relevant to its factor and all five factors are significantly relevant to each other and to the conceptual structure.

In terms of discriminant validity, the BES performed well. In agreement with previous study [7], we found a higher prevalence of breastfeeding difficulties in primiparous mothers.

Concurrent validity was also confirmed by the moderate correlations between the scores of BES and GHQ-28. Previous study revealed that among mothers who experienced poor support, breastfeeding difficulties might lead to depression during the first 6 months postpartum [7]. Surprisingly, mothers with higher education were more likely to experience breastfeeding difficulties. Qualitative studies are needed to answer why mothers with higher education express more breastfeeding difficulties than others in Iran.

Considering the predictive validity of the BES, we found that the BES could predict the continuation of EBF at the first month postpartum, which is in agreement with the results of previous studies, which were not using the BES to measure breastfeeding difficulties [6, 10, 11].

Internal consistency of the first 18 items of the BES was satisfactory (0.83) which was comparable with Wambach's study that found that the *α*-coefficient for the 18 items at 3, 6, and 9 weeks postpartum was 0.77, 0.77, and 0.81, respectively [13]. However, our results showed that the *α*-coefficient for the one subscale of the new BES was lower than 0.6 (process subscale, 0.38). These results were comparable to those in the study of Wambach [13] in which the *α*-coefficient for two subscales at 6 weeks postpartum was lower than 0.6. Since the value of alpha depends on the number of items on a scale, it is a common observation that *α*-coefficient decreases when the number of items decreases [30]. The item-subscale analysis showed that there was no item-subscale correlation coefficient lower than 0.2 and all interitem correlation coefficients were less than 0.80 and higher than zero, indicating satisfactory reliability.

In this study, we adapted the original English version of the BES to Farsi. The results of this study show that the Farsi version of the BES is a reliable and valid instrument for measuring breastfeeding difficulties in Iranian mothers. We recommend that further studies be designed to identify cut-off point for the BES in the first weeks postpartum for the task of screening for breastfeeding discontinuation. In addition, we recommend that in future studies the two five-factor models be tested to examine and compare their structures.

### 4.1. Implications for Practice and Policy

Providers of obstetric care should pay more attention to mothers having difficulty with breastfeeding during early postpartum period and consider screening for breastfeeding difficulties in early postpartum period. The Farsi version of the BES can be used as a part of routine assessments in the postpartum period and will fill an important gap in measuring breast feeding difficulties in mothers in the postpartum period in Iran.

### 4.2. Limitations

We did not assess reliability through test-retest analysis because the nature of breastfeeding difficulties is transient during the first months postpartum.   Our sample consisted of multiparas and primiparas. Since parity is an important factor to express breastfeeding difficulties, it is likely that results improved if we made study on a larger sample of primiparas. The results are limited to mothers in early postpartum period and cannot be generalized to late postpartum period.

## 5. Conclusion

The present study confirmed the content validity of the BES. In addition, reliability and construct validity of the Farsi version of the first 18 items of the BES were confirmed. Although a new five-factor model was proposed, the original structure was not rejected. Further studies are needed to compare the two five-factor structures of the BES.

## Figures and Tables

**Table 1:** Results of exploratory factor analysis (EFA).

Item	Factors				
	1	2	3	4	5
Q16: feeling tense and overwhelmed	**0.819**	0.178	0.095	0.045	0.089
Q18: difficulty in combining work and breastfeeding	**0.751**	0.171	0.105	0.067	0.163
Q12: feeling very tired	**0.716**	0.247	0.207	0.026	0.257
Q14: difficulty in positioning baby	**0.488**	0.236	0.352	0.177	−0.250
Q17: feeling embarrassed when nursing	**0.440**	0.117	0.302	−0.075	−0.304
Q10: worry of not having enough milk	0.189	**0.821**	0.137	0.038	0.073
Q13: worry that baby was not getting enough milk	0.195	**0.772**	0.204	0.059	−0.016
Q15: worry about baby's weight gain	0.209	**0.630**	0.117	0.085	−0.006
Q5: baby reluctant to nurse due to sleepiness	0.107	0.108	**0.738**	−0.039	0.119
Q6: baby reluctant to nurse due to fussiness	0.134	0.079	**0.711**	−0.040	0.003
Q11: baby having difficulty in sucking	0.351	0.242	**0.574**	0.221	−0.052
Q4: baby having difficulty in latching on	0.198	0.313	**0.568**	0.330	0.086
Q1: sore nipple	0.106	0.050	−0.145	**0.866**	0.028
Q2: cracked nipple	0.041	0.178	0.011	**0.844**	−0.023
Q3: breast engorgement	0.161	0.002	0.132	**0.553**	0.294
Q7: breast infection	−0.165	−0.030	0.241	**0.397**	0.083
Q8: leaking breasts	0.176	−0.233	0.196	0.198	**0.689**
Q9: baby nursing too frequently	0.078	0.351	−0.037	0.068	**0.688**
Eigenvalue^b^	2.592	2.246	2.191	2.186	1.327
Variance^b^	14.402	12.478	12.171	12.145	7.374

Factors: 1: mother concern, 2: insufficient milk concern, 3: baby concern, 4: breast concern, and 5: process concern.

Item numbers refer to question numbers in the original questionnaire. ^b^The percentage variance and eigenvalues explained for rotated factor matrices. Extraction method: principal component analysis, and rotation method: varimax with Kaiser normalization.

**Table 2:** Results of confirmatory factor analysis (CFA) with 18 items.

Model	Chi-square	*P*	Chi-square/df	RMSEA	SRMR	CFI
As originally assigned by Wambach	476	0.00	3.8	**0.09**	0.072	0.90
The model of this study	301	0.00	2.4	0.064	0.064	0.95

Observation below the recommended value is shown in bold character. Chi-square/df: minimum fit function/degree of freedom; RMSEA: root mean square error of approximation; SRMR: standardized root mean square residual; CFI: comparative fit index.

**Table 3:** Cronbach's alpha of the BES subscales as originally assigned by Wambach.

Subscales^†^	Cronbach's alpha			
	Wambach's study [13]	As originally assigned by Wambach	Primiparas	Multiparas
Mechanic	0.60 (5 items)	0.73 (5 items)	0.79	0.53
Insufficient milk	0.86 (3 items)	0.74 (3 items)	0.78	0.68
Breast	0.68 (3 items)	0.66 (3 items)	0.70	0.53
Social	0.48 (2 items)	0.40 (2 items)	0.50	0.16
Process	0.56 (5 items)	0.54 (5 items)	0.58	0.49

Total	0.77	0.83	0.86	0.71

^†^Subscales as originally assigned by Wambach (1998) [13]; the order of the subscales is based on the order of the factors extracted by Wambach.

**Table 4:** Descriptive statistics and Cronbach's alpha for the BES subscales as extracted by the EFA.

Subscales	Mean (SD)	Minimum	Maximum	Cronbach's alpha		
				All	Primiparas	Multiparas
Mother	8.52 (3.26)	5	21	0.76	0.80	0.71
Insufficient milk	5.53 (2.52)	3	14	0.74	0.78	0.68
Baby	5.85 (2.42)	4	19	0.72	0.75	0.60
Breast	6.21 (2.65)	4	20	0.65	0.68	0.60
Process	5.29 (1.73)	2	10	0.38	0.38	0.38

Total scale	31.40 (8.51)	18	74	0.83	0.86	0.71

The order of the subscales is based on the order of the factors extracted by the EFA.

## References

[1] Ip S, Chung M, Raman G (2007). Breastfeeding and maternal and infant health outcomes in developed countries. *Evidence Report/Technology Assessment*.

[2] World Health Organization (2002). Infant and young child nutrition; global strategy for infant and young child feeding.

[3] Olang B, Farivar K, Heidarzadeh A, Strandvik B, Yngve A (2009). Breastfeeding in Iran: prevalence, duration and current recommendations. *International Breastfeeding Journal*.

[4] http://www.unicef.org/infobycountry/iran_statistics.html.

[5] Mehrparvar S, Varzandeh M (2011). lnvestigation of decreasing causes of exclusive breastfeeding in children below six months old, in Kerman City during 2008-2009. *Journal of Fasa University of Medical Sciences*.

[6] Gerd A-T, Bergman S, Dahlgren J, Roswall J, Alm B (2012). Factors associated with discontinuation of breastfeeding before 1 month of age. *Acta Paediatrica, International Journal of Paediatrics*.

[7] Chaput KH (2013). *The effect of breastfeeding difficulty and associated factors on postpartum depression [Ph.D. thesis]*.

[8] Giugliani ERJ (2004). Common problems during lactation and their management. *Jornal de Pediatria*.

[9] Mozingo JN, Davis MW, Droppleman PG, Merideth A (2000). It wasn’t working: women’s experiences with short-term breastfeeding. *MCN. The American Journal of Maternal Child Nursing*.

[10] Palmer L, Carlsson G, Mollberg M, Nystrom M (2012). Severe breastfeeding difficulties: existential lostness as a mother-women's lived experiences of initiating breastfeeding under severe difficulties. *International Journal of Qualitative Studies on Health and Well-Being*.

[11] Scott JA, Binns CW, Oddy WH, Graham KI (2006). Predictors of breastfeeding duration: evidence from a cohort study. *Pediatrics*.

[12] Wambach K, Campell SH, Gill SL, Dodgson JE, Abiona TC, Heinig MJ (2005). Clinical lactation practice: 20 years of evidence. *Journal of Human Lactation*.

[13] Wambach KA (1998). Maternal fatigue in breastfeeding primiparae during the first nine weeks postpartum. *Journal of Human Lactation*.

[14] Taveras EM, Capra AM, Braveman PA, Jensvold NG, Escobar GJ, Lieu TA (2003). Clinician support and psychosocial risk factors associated with breastfeeding discontinuation. *Pediatrics*.

[15] Wambach K Development of an instrument to measure breastfeeding outcomes: The Breastfeeding Experience Scale.

[16] Wambach KA (2003). Lactation mastitis: a descriptive study of the experience experiencia. *Journal of Human Lactation*.

[17] Wambach KA (1997). Breastfeeding intention and outcome: a test of the theory of planned behavior. *Research in Nursing & Health*.

[18] Goldberg DP, Hillier VF (1979). A scaled version of the general health questionnaire. *Psychological Medicine*.

[19] Ebrahimi A, Molavi H, Moosavi G, Bornamanesh A, Yaghobi M (2007). Psychometric properties and factor structure of general health questionnaire 28 (GHQ-28) in Iranian psychiatric patients. *Journal of Research in Behavioural Sciences*.

[20] Gross J, Wambach K, Aaronson L Perceived Behavioral Control of Breastfeeding [BSN Honors Research1:1].

[21] Streiner D, Norman G (1995). *Health Measurement Scales: A Practical Guide To Their Development and Use*.

[22] Dixon J, Monro BH (2001). Factor analysis. *Statistical Methods for Health Care Research*.

[23] Waltz CF, Strickland OL, Lenz ER (2010). *Measurement in Nursing and Health Research*.

[24] Bentler PM, Bonett DG (1980). Significance tests and goodness of fit in the analysis of covariance structures. *Psychological Bulletin*.

[25] Hu L-T, Bentler PM (1999). Cutoff criteria for fit indexes in covariance structure analysis: conventional criteria versus new alternatives. *Structural Equation Modeling*.

[26] Kline RB, Petscher Y, Schatsschneider C (2013). Exploratory and confirmatory factor analysis. *Applied Quantitative Analysis in the Social Sciences*.

[27] Colton D, Covert RW (2007). *Designing and Constructing Instruments for Social Research and Evaluation*.

[28] Lawshe C (1975). A quantitative approach to content validity. *Personnel Psychology*.

[29] Polit D, Beck C (2004). *Nursing Research: Principles and Methods*.

[30] Field A (2009). *Discovering Statistics Using SPSS*.

